# College Students and Nature: Differing Thoughts of Fear, Danger, Disconnection, and Loathing

**DOI:** 10.1007/s00267-019-01172-9

**Published:** 2019-05-10

**Authors:** Dorceta E. Taylor

**Affiliations:** School for Environment and Sustainability, University of Michigan, Ann Arbor, MI USA

**Keywords:** Connectedness, Disconnection, Whites, Blacks, ASIANS, LatinX

## Abstract

Despite the existence of a robust body of research that investigates human–nature connections, few scholars have examined what people tend to ponder when they think of nature. The objective of the study is to find out how college and university students think about nature. The study also seeks to identify which factors are most significant in influencing students’ thoughts about nature. This paper analyzes racial, gender, class, and academic differences in the way college students think about nature. The study of 287 American students found that respondents thought about a wide range of concepts and ideas when they contemplate nature. This article focuses on the demographic differences in thoughts about fear, danger, and loathing. This set of ideas has been the subject of scholarly research, and the findings presented herein contribute to this body of scholarship. The paper discusses both descriptive and multivariate techniques that are used to explore the topic. The study found that white students are less likely than racial/ethnic minorities to think about disconnection, predators, getting lost, loathsome or hateful places, fear, and danger when they think of nature. However, the results also show that it would be inaccurate to describe racial/ethnic minorities as universally fearful of and disconnected from nature. Moreover, the paper demonstrates that race is not the only explanatory variable that has significant impacts in multivariate models—the student’s academic interest has significant impacts on thoughts about natural hazards, disconnection, predators, human-made hazards, and loathsome or hateful places. Gender, age, parental education, and first-generation college attendance also has significant impacts on the dependent variables.

## Introduction

Nature occupies an essential space in American environmental discourses. Since the 19th century, activists and professionals have extolled the virtues of nature and sought to protect and manage it. Contemporary environmental managers steward natural areas and encourage public participation in conservation activities. Despite the importance of public engagement in nature protection, we know little about what people think about when they think of nature. The paucity of knowledge is particularly glaring when it comes to what we know of young people's thoughts about nature. Very few studies have explored what comes to mind when young adults reflect on nature.

There is general agreement that nature is vital to human well-being, but, what do people associate nature with when they think of it? What are the differences and similarities in the way people think of nature? This line of inquiry is significant as deeper insights into the ways people think about nature can foster more sustainable and meaningful human–nature interactions. These insights can also help us understand differing responses to nature. Hence, this paper will examine how American college students think about nature. Given the wide variety of ideas that surface when cogitating nature, the article will focus specifically on conceptions related to fear, danger, alienation, and loathing. It will also analyze how race, gender, class, the participant’s educational attainment, and parental educational background are related to such thoughts.

### The Connectedness-to-Nature Conceptual Framework

Research on connectedness to nature is relevant to this study. For decades, scholars using biological and environmental psychology frameworks have theorized about human–nature relationships. For instance, Wilson (1993; 1984) posits a biophilia hypothesis that asserts that humans have an innate connection to nature. Adherents of this thesis contend that the human–nature connection is mediated by values, and those values are rooted in biology or our evolutionary past (Kaplan 1992; Kellert 1993; Mayer et al. 2009; Ulrich 2008; 1993). The biophilia thesis has led some researchers to attempt to measure people's emotional connection or kinship to the natural world (Mayer and Frantz 2004; Olivos et al. 2011; Perrin and Benassi 2009). Mayer and Frantz (2004) and Pereira and Forster (2015) argue further that connectedness to nature is a strong predictor of ecological behavior. Dutcher et al. (2007) also claim that environmental values stem from a sense of connectivity to nature. The researchers argue further that connectivity with nature has a significant, positive relationship with environmental concern and environmental behavior.

These arguments are significant since there is a considerable body of research that portrays blacks as being disconnected from nature. This line of research also suggests that because of their race and evolutionary past, blacks have unique outdoor recreational preferences. For instance, Kellert (1984) and Schroeder (1989) argue that blacks and urban dwellers are less likely to be attracted to nature and are less knowledgeable about environmental issues than white suburbanites or white rural residents.

Similarly, Johnson (1998) claims that blacks have an aversion to wildlands. She argues that the alienation is a result of the collective memory of slavery, lynching, and other acts of violence visited on African Americans in the woods. Johnson, who studied 147 whites and 116 African Americans in Gadsen County, Florida, found that race was a strong predictor of attachment to wildlands. The study revealed that blacks were less likely than whites to have strong attachments to wildlands. Lewis and Hendricks (2006) also report that blacks are isolated from the forests.

Some researchers studying outdoor leisure pursuits and affinity toward natural landscapes argue that blacks are disconnected from nature because they have a deep-rooted dread about undisturbed natural and wild spaces. Scholars contend that fear leads to discomfort and avoidance of wildlands. For instance, Talbot and Kaplan (1984) argue that black Detroiters prefer manicured urban settings over undisturbed or minimally disturbed wooded settings. The researchers surmise that blacks favor the open manicured settings over densely wooded landscapes because they are frightened of the woods and believe it is dangerous. Although some scholars challenge the assumptions that leisure behavior or landscape preferences are a function of genetics, evolution, adaptation, and are “hardwired”, (Joye and Van den Berg 2011; Blanchette 2006) the biophobia thesis persists in the literature (for instance, see Ulrich 1993).

Other researchers have also examined the issue of fear in park usage and interactions with nature. In this vein, Gobster (2002) conducted a study of visitors in Chicago's Lincoln Park and found that blacks were less likely than whites to say they preferred the park's natural attributes over developed facilities, but LatinX and Asians recreators put a premium—to an equal or greater extent than whites—on the park’s naturalistic features. Although research, already discussed above, hypothesizes or finds that blacks fear being in natural settings, in the Lincoln Park study it was whites who were most concerned about safety. White park users were more than twice as likely as ethnic minority park users to fear for their safety.

Brownlow (2006) studied the Fairmount Park System in Philadelphia and found what he describes as a “legacy of fear towards the city's natural environment” that has significant and lingering impacts on African American women. Hyun (2005) suggests that the fear of nature is transmitted intergenerationally.

However, several researchers challenge the assumption that race is the pre-eminent determinant of connections to nature and landscape preferences (Taylor 2018; Carr and Williams 1993; Shinew et al. 1996). Shinew et al. (1996) argue that class influences people's perceptions of nature, whereas Virden and Walker (1999) contend that gender is also influential in landscape preferences.

### Focus on Students

A limited number of studies have examined connectedness to nature and landscape preferences among American students. Peterson (1977) conducted a study of preference for scenery and reported that black high school students preferred urban landscapes, while white students were more attracted to pastoral settings. Similarly, Medina (1983) claims that black youths preferred urban scenes while the environmental educators to whom they were being compared preferred natural areas with minimal or no disturbance.

Bixler et al. (2004) explored urban students' fear and discomfort in wildland areas in their research by asking 48 naturalists to assess how students responded to nature while on field trips. The naturalists reported that students feared the animals, trees, and people while exploring the wilds. In 1997, Bixler and Floyd studied 450 suburban and rural 8 graders in Texas and concluded that many of the students were fearful of and disgusted by wildlands. The researchers found that strong fear and disgust was associated with an aversion to wild landscapes. Floyd et al. (1995) studied 1200 black and white middle and high school students and reported that white students tended to rate wildland activities more highly than blacks. The scholars found that fear was a factor in the students' ratings. That is, fear of nature and preference for urban environments were positively related to a desire to participate in social activities that did not occur in wildlands.

Despite being a large and relatively influential segment of the adult population, few studies have focused on college or university (hereinafter college) students. Virden and Walker (1999) studied 323 students attending a public university in the western United States and found that white and LatinX students were more likely to prefer remote, less developed settings than blacks. In the end, Virden and Walker concluded that the white participants in their study “considered a forest to be safer than black and LatinX respondents, who perceived it as more threatening” (p. 233).

Manning (2012) studied how students at Southern Utah University were connected to nature and found that male students scored higher on the connectedness-to-nature scale than females. He also stated that urban students had higher scores on the scale than suburban and rural students. Lakenau (2018) studied university students too and found that an introductory ecology course enhanced students' connectedness to nature. However, Nisbet et al. (2011), who studied college students enrolled in semester-long environmental courses, did not witness any changes in the level of students' connectedness to nature that were attributable to taking the courses.

### Thoughts About Nature

Despite the plethora of studies that examine people’s connections to nature and landscapes, researchers usually do not ask study participants what they think of when they think about nature. Moreover, studies do not examine how frequently respondents think about different aspects of nature while reflecting on the topic. Because there are unexplored connections between people's thoughts and attitudes about nature, Johansson and Henningsson (2011) suggest that attitudes are related to structures of thought, hence, we should pay more attention to what people think about nature. Bang et al. (2007) and Fischer and Young (2007) argue further that attitudes are vital components of mental constructs about the natural environment. Consequently, researchers have used word-association techniques to find out what thoughts study participants associate with nature (Buijs and Elands 2013; Fischer and Young 2007; Taylor 2018).

A few researchers have explored how students think about nature; scholars have also analyzed racial differences in students’ thoughts about nature. For instance, Aron and Witt (2011) studied urban minority youths and found that when it came to nature, they were fearful of wild animals, the unknown, and were also anxious about lack of comfort and convenience.

Taylor (2018) studied 157 college students and asked them to say what came to mind when they thought about nature. Respondents identified 47 discrete ideas and concepts. The study, which examined racial differences in thoughts about nature and landscape preferences, found that black, white, and other minority students identified specific objects (object-specific fear) and situations (situational fear) in natural settings that they feared, but did not express a generalized fear of nature. In essence, black and other minority students in the study did not express the widespread alienation, aversion, and disgust for nature that other studies have reported.

Other research involving students have also found that maturity—measured in years of education—impacts students’ perception of nature (Rajeski 1982; Strommen 1995). Besides, Gotch and Hall (2004) found that family members influenced how teens perceived nature. Hyun (2005) corroborates this finding.

## Methods

### Study Objectives and Survey Description

The objective of the study is to find out how college and university students think about nature. The study also seeks to identify which factors are most significant in influencing students’ thoughts about nature. This study builds on the work of Buijs and Elands (2013) and Taylor (2018) in exploring what people think about when they contemplate nature. To conduct their study of how environmental professionals and lay people thought about nature, Buijs and Elands (2013), used a word-association technique to identify the implicit meanings study participants attach to or associate with the stimulus term, “nature.” The word association technique has also been used by environmental researchers such as Aaron and Witt (2011) to study urban youth’s definitions and perceptions of nature.

Taylor (2018) asked respondents in her study what came to their mind when they thought about nature. They provided open-ended responses to the question. The answers varied in length from short phrases to lengthy paragraphs. Hence, some responses generated multiple concepts. Each unique idea and concept identified was coded and analyzed.

The study analyzed in this paper uses a survey to produce both the qualitative and quantitative analyses presented below. The survey, designed and administered on a Qualtrics platform, asked respondents the following question: When you hear the word nature, which of the following comes to mind? Respondents in the current study got a list of words or short phrases that participants in past studies associated with nature. These concepts were generated from Taylor’s (2018) study as well as from the scholarly literature related to connectedness to nature (Buijs and Elands 2013; Aaron and Witt 2011; Dutcher et al. 2007).

In the research presented here, each nature-related concept has a five-point Likert scale, on which study participants indicated how often they thought about each aspect of nature. The scale points are: 1 = never, 2 = seldom, 3 = about half the time, 4 = most of the time, 5 = always. Hence, respondents were instructed to choose one answer on the scale for each concept to indicate how frequently each thought comes to mind.

Likert scales are commonly used in analyses of this type to assess the presence or absence of a phenomenon and frequency of occurrence. In this case, it accomplishes both goals: (a) it is used to identify whether respondents think about a concept related to nature, and (b) if they do so, how often do those thoughts occur. Similar Likert scales have been used in environmental research by scholars such as Bunyan et al. (2016) in a study of citizen’s responses to environmental public goods and coastal flooding in the UK. Dutcher et al. (2007) also used Likert scales in their study of Pennsylvania landowners’ connectivity with nature and environmental values. The survey also contained questions that collected information on the race, gender, age, educational attainment, academic interest, and social class of each respondent.

### Sample and Response

College and university students participating in science, technology, engineering, math (STEM), as well as arts, humanities, and social sciences courses and programming were asked to participate in the study. The study used a purposive sampling technique. Purposive sampling is appropriate for a study of this nature. It is a nonprobability data collection technique used to study population subgroups and identify and select informants who are knowledgeable about or have experience with the subject area of interest. The method is also suitable for making comparisons between cases (Etikan et al. 2016; Palinkas et al. 2015; Ford et al. 2009; Teddlie and Yu 2007; Guarte and Barrios 2006; Schreuder et al. 2001). It is appropriate for qualitative and quantitative analyses (Tongco and Dolores 2007). The purposive sampling techniques widely used in social science (Teddlie and Yu 2007; Guarte and Barrios 2006), mental health (Palinkas et al. 2015) ethnobotany (Tongco and Dolores 2007), and environmental research (Ford et al. 2009; Schreuder et al. 2001; Hsu 2009).

The students mentioned above were contacted to ensure that the sample contained participants with varying levels of familiarity with nature and the environment. This approach ensures that comparisons are feasible. It also limits selection biases and allows the researcher to apply the findings beyond the sample.

Seven hundred students attending public and private colleges and universities (after this college) in the Northeast, Mid-Atlantic, Midwest, South, Southeast, Southwest, Mountain, and Pacific regions of the country were asked to participate in the study. I administered the survey from April 28, 2017, through June 13, 2017. In all, 287 students submitted usable responses. The response rate for the study is 41%. The response rate is robust, as response rates for electronic surveys are usually below 30% (Sax et al. 2003; Kaplowitz et al. 2004; Buijs and Elands 2013).

The sample contains 102 whites, 63 Asians, 62 blacks, 47 LatinX, and 13 others (these are students of Native American, Pacific Islander, Arab, and Middle Eastern ancestry). For this analysis, LatinX and other students are combined to create a category robust enough to withstand multivariate analysis.

There were 221 females and 66 males in the sample. Respondents ranged in age from 18 to 46 years old. However, 246 of the students were less than 25 years of age. Fifty-four (18.8%) of the study participants were graduate students, while 233 were undergraduates. Just over half of the students (151) were majoring in the natural and physical sciences. Eighty-six were social science, humanities, and arts majors, while 50 were in engineering, math, computer science, or their majors were unknown or undecided.

Respondents were asked to say what the educational attainment of their parents or guardians were since parental/guardian educational attainment provides an indicator of social class. One hundred and eighteen (41.1%) of the study participants indicated that their parents/guardians had not attended college. Therefore, 169 or 58.9% of the students in the study were from households with college-educated parents/guardians. I obtained data on two other indicators of social class—Pell Grant eligibility and first generation in college. Pell Grants are means-tested financial assistance awarded to low-income students. One hundred and twenty-seven (44.3%) of the samples were eligible for Pell Grants. Roughly, a third of the sample were first-generation college students.

### Data Analysis

Respondents assessed 58 descriptors or concepts relating to nature. For analytical purposes, I organized the descriptors into eight typologies or thematic areas (see Table 1 and Box 1). Though there are 58 categories of nature thoughts, this paper will analyze the responses of one topical area—i.e., thoughts related to fear, danger, and loathing. Loathing describes ideas related to hating or despising nature. In Taylor (2018), respondents who indicated that they loathed nature expressed hatred and a strong dislike for nature.

**Table 1:** What students think about when they contemplate nature

Thoughts about nature	Total sample		Infrequently		Frequently	
	Number	Mean	Number	Percent	Number	Percent
*1. Forests, green spaces, and creatures*						
The outdoors	285	4.65	9	3.2	276	96.8
Trees, forests, plants	287	4.58	14	4.9	273	95.1
Green space	281	4.21	19	6.8	262	93.2
Animals	286	4.04	27	9.4	259	90.6
Flowers	286	3.84	47	16.4	239	83.6
Parks	286	3.35	82	28.7	204	71.3
*2. Landscapes*						
Wild, wilderness, untamed landscapes	285	4.21	24	8.4	261	91.6
Undisturbed landscapes	285	3.97	39	13.7	246	86.3
Spaces needing protection/preservation	285	3.84	43	15.1	242	84.9
Remote, far away landscapes	286	3.60	54	18.9	232	81.1
Rural landscapes	284	3.09	96	33.8	188	66.2
Urban landscapes	285	2.12	202	70.9	83	29.1
*3. Ecosystems and species interactions*						
The natural world	284	4.20	29	10.2	2.55	89.8
Ecosystems, biomes	283	3.95	46	16.3	237	83.7
Biodiversity	285	3.51	76	26.7	209	73.3
Species interactions	285	3.49	81	28.4	204	71.6
Human interactions with natural systems	284	3.21	102	35.9	182	64.1
Endangered species	284	3.00	117	41.2	167	58.8
*4. Physical features*						
Water bodies, oceans, lakes, rivers	284	4.37	14	4.9	270	95.1
Mountains, hills	286	4.15	34	11.9	252	88.1
Grasslands	285	3.63	51	17.9	234	82.1
Soils	286	3.42	91	31.8	195	68.2
Beaches	285	3.25	99	34.7	186	65.3
Rocks	287	3.17	105	36.6	182	63.4
Deserts	286	2.94	127	44.4	159	55.6
Non-living things	285	2.82	130	45.6	155	54.4
Tundra	285	2.58	156	54.7	129	45.3
*5. Elements*						
Fresh air	286	4.05	56	19.6	230	80.4
Sun, sunshine	286	3.74	57	19.9	229	80.1
Energy	285	3.59	73	25.6	212	74.4
Wind	283	3.47	79	27.9	204	72.1
*6. Utility*						
Sustains life	286	3.56	69	24.1	217	75.9
The environments we live in	281	3.43	78	27.8	203	72.2
Provides food, sustenance	287	3.43	80	27.9	207	72.1
Recreation	283	3.23	84	29.7	199	70.3
Learning from nature	285	3.23	98	34.4	187	65.6
*7. Sensations, feelings, and experiences*						
Peaceful, tranquil	286	4.24	19	6.6	267	93.4
Scenic, beautiful	286	4.28	27	9.4	259	90.6
Happiness	286	3.83	52	18.2	234	81.8
Quiet, still	284	3.75	48	16.9	236	83.1
Welcoming spaces	285	3.71	54	18.9	231	81.1
Therapeutic	285	3.69	54	18.9	231	81.1
Freedom	285	3.60	69	24.2	216	75.8
Lack of humans	286	3.59	51	17.8	235	82.2
Safe space, refuge	286	3.42	85	29.7	201	70.3
Wonderment	281	3.35	90	32.0	191	68.0
Spirituality	286	3.33	88	30.8	198	69.2
Connected	285	3.32	82	28.8	203	71.2
Nurturing spaces	284	3.18	100	35.2	184	64.8
Mysterious, intriguing	283	3.06	112	39.6	171	60.4
*8. Fear, danger, and loathing*						
Natural hazards	286	2.72	146	51.0	140	49.0
Predators	284	2.64	152	53.5	132	46.5
Disconnected	284	2.53	151	53.2	133	46.8
Getting lost	285	2.53	159	55.8	126	44.2
Human-made hazards	286	2.27	183	64.0	103	36.0
Dangerous	285	1.91	225	78.9	60	21.1
Fearful	284	1.90	221	77.8	63	22.2
Loathsome, hateful places	285	1.85	220	77.2	65	22.8

#### Box 1. Major topical areas of thoughts about nature

1. Forests, green spaces, and creatures
2. Landscapes
3. Ecosystems and species interactions
4. Physical features
5. Elements
6. Utility
7. Sensations, feelings, and experiences
8. Fear, danger, and loathing

It is beyond the scope of a single-journal article to analyze all 58 descriptors, hence the focus on one typology. The thematic area that deals with fear, danger, and loathing contains eight concepts, these are the dependent variables analyzed in this paper (Box 2, Table 2). Examining this nexus of ideas is appropriate because scholars and environmental managers have expressed keen interest in the racial and gender differences reported in earlier studies. This topical area is fascinating because of the racialization of the discourse related to people’s connection to nature. That is, blacks and other ethnic minorities have been linked to negative perceptions of nature (Kellert 1984; Schroeder 1989; Johnson 1998; Lewis and Hendricks 2006; Talbot and Kaplan 1984; Brownlow 2006; Peterson 1977; Medina 1983; Bixler et al. 2004; Bixler and Floyd 1997; Virden and Walker 1999).

#### Box 2. Dependent variables—concepts related to fear, danger, and loathing

1. Natural hazards
2. Disconnected
3. Predators
4. Getting lost
5. Human-made hazards
6. Loathsome, hateful places
7. Fearful
8. Dangerous

**Table 2:** Dependent variables, definitions, and percentage

Dependent variables and definitions	Frequency	Percentage
*Natural hazards*		
Never	54	18.9
Seldom	92	32.2
About half the time	56	19.6
Most of the time	48	16.8
Always	36	12.6
*Disconnected*		
Never	85	29.9
Seldom	66	23.2
About half the time	58	20.4
Most of the time	48	16.9
Always	27	9.5
*Predators*		
Never	52	18.3
Seldom	100	35.2
About half the time	61	21.5
Most of the time	40	14.1
Always	31	10.9
*Getting lost*		
Never	53	18.6
Seldom	106	37.2
About half the time	64	22.5
Most of the time	46	16.1
Always	16	5.6
*Human-made hazards*		
Never	116	40.6
Seldom	67	23.4
About half the time	39	13.6
Most of the time	38	13.3
Always	26	9.1
*Loathsome, hateful places*		
Never	171	60.0
Seldom	49	17.2
About half the time	19	6.7
Most of the time	30	10.5
Always	16	5.6
*Fearful*		
Never	132	46.5
Seldom	89	31.3
About half the time	35	12.3
Most of the time	14	4.9
Always	14	4.9
*Dangerous*		
Never	118	41.4
Seldom	107	37.5
About half the time	39	13.7
Most of the time	11	3.9
Always	10	3.5

Environmental managers are curious about this. They want to know how to manage natural areas so that they can engage the public, introduce more people to new experiences, get more significant input from lay people, and foster an ethic of stewardship for nature. This research will help to shine a spotlight on how one segment of the population thinks about nature. It is a first step toward assisting managers in understanding how to garner greater public interest and engagement in nature-related issues.

All the data are coded and analyzed in SPSS 24. As mentioned before, respondents were asked to use a five-point scale to indicate how frequently they thought about each concept or idea. The first level of analysis examined the distribution of responses on this scale (Table 1). Mean scores, calculated on the uncollapsed values of the descriptors, are reported. They range a low of 1 to a high of 5.

Because the sample size is relatively small, it is not feasible to conduct multivariate analyses on variables that have five response categories. Consequently, the five groups were collapsed to two, thereby converting these variables into binary-dependent variables. The two response categories analyzed for each dependent variable is: never/seldom and about half the time/most of the time/always. For ease of reading, never/seldom will be referred to as infrequently, and about half the time/most of the time/always will be referred to as frequently (the terms regularly or often are also used). Researchers such as Bunyan et al. (2016) use the technique of converting dependent variables to binary forms in their study of attitudes toward the climate risk.

The article examines eight independent variables (Box 3); the definitions and the categorical distributions appear in Table 3. Several researchers, discussed above, have reported that race is an essential factor in nature preference. They argue that blacks have an aversion to and fear of natural settings and a preference for urban landscapes (Kellert 1984; Schroeder 1989; Johnson 1998; Lewis and Hendricks 2006; Talbot and Kaplan 1984; Brownlow 2006; Peterson 1977; Medina 1983; Bixler et al. 2004; Bixler and Floyd 1997; Virden and Walker 1999). However, Taylor (2018) provides evidence to the contrary.

#### Box 3. Independent variables

1. Race
2. Gender
3. Age
4. Student status
5. Academic major
6. Parent/guardian’s education
7. Pell Grant eligible
8. First-generation college student

**Table 3:** Sociodemographic variables, definitions, and percentage

Explanatory variables and definitions	Frequency	Percentage
*Race*		
White or Caucasian (not LatinX)	102	35.5
Asian	63	22.0
Black or African American	62	21.6
LatinX/other	60	20.9
*Gender*		
Male	66	23.0
Female	221	77.0
*Age*		
18–24 years	246	85.7
25–46 years	41	14.3
*Educational attainment*		
Graduate student	54	18.8
Undergraduate	233	81.2
*Major*		
Natural or physical sciences	151	52.6
Social sciences, humanities, arts	86	30.0
Engineering, math, computer science, unknown	50	17.4
*Parental/guardian education*		
Parents/guardians are not college educated	118	41.1
Parents/guardians are college educated	169	58.9
*Federal Pell Grant (available to low-income students)*		
Not eligible for Pell Grant	160	55.7
Federal Pell Grant eligible	127	44.3
*First-generation college student*		
Not a first-generation college student	193	67.2
First-generation college student	94	32.8

Prior research has also shown that age (Bunyan et al. 2016; Tjernstrom and Tietenberg 2008; Kellstedt et al. 2008), gender (Virden and Walker 1999); educational attainment (Rajeski 1982; Strommen 1995; Tjernstrom and Tietenberg 2008), income or social class (Shinew et al. 1996; Tjernstrom and Tietenberg 2008), and relatives (Gotch and Hall 2004; Hyun 2005) can influence environmental attitudes. Studies have also found that females perceive the environment as riskier than males (Kellstedt et al. 2008; van der Linden 2015; Bunyan et al. 2016). Manning (2012) also found that males were more connected to nature than females.

There was no evidence of multicollinearity between the independent variables. The variance inflation factor (VIF) was well below 5—a suggested threshold—for all tests performed. The paper will discuss both descriptive and multivariate analyses. Cross-tabulations were conducted to facilitate a descriptive analysis of how each independent variable was related to the dependent variables (Table 4).

**Table 4:** Cross-tabulations of dependent and explanatory variables

Explanatory variables and categories	Frequency	Percentage															
		Natural hazards		Disconnected		Predators		Getting lost		Man-made hazards		Loathsome or hateful		Fearful		Dangerous	
		Infrequently	Frequently	Infrequently	Frequently	Infrequently	Frequently	Infrequently	Frequently	Infrequently	Frequently	Infrequently	Frequently	Infrequently	Frequently	Infrequently	Frequently
*Race*																	
White or Caucasian (not LatinX)	102	51.5	48.5	64.4	35.6	67.3	32.7	71.3	28.7	66.3	33.7	92.1	7.9	86.0	14.0	90.1	9.9
Asian	63	50.8	49.2	48.4	51.6	52.4	47.6	47.6	52.4	73.0	27.0	76.2	23.8	77.8	22.2	73.0	27.0
Black or African American	62	53.2	46.8	47.5	52.5	36.1	63.9	47.5	52.5	62.9	37.1	67.2	32.8	75.4	26.4	72.1	27.9
LatinX/other	60	48.3	51.7	45.0	55.0	49.2	50.8	46.7	53.3	51.7	48.3	63.3	36.7	66.7	33.3	73.3	26.7
*Gender*																	
Male	66	43.1	56.9	39.1	60.1	53.8	46.2	53.2	47.7	58.5	41.5	69.2	30.8	73.4	26.6	72.3	27.7
Female	221	53.4	46.6	57.3	42.7	53.4	46.6	56.8	43.2	65.6	34.4	79.5	20.5	79.1	20.9	80.9	19.1
*Age*																	
18–24 years	246	54.3	45.7	53.9	46.1	54.9	45.1	56.6	43.4	66.1	33.9	78.7	21.3	78.6	21.4	77.9	22.1
25–46 years	41	31.7	68.3	48.8	51.2	45.0	55.0	51.2	48.8	51.2	48.8	68.3	31.7	73.2	26.8	85.4	14.6
*Educational attainment*																	
Graduate student	54	38.9	61.1	42.6	57.4	41.5	58.5	48.1	51.9	53.7	46.3	68.5	31.5	74.1	25.9	85.2	14.8
Undergraduate	233	53.9	46.1	55.7	44.3	56.3	43.7	57.6	42.4	66.4	33.6	79.2	20.8	78.7	21.3	77.5	22.5
*Major*																	
Natural or physical sciences	151	43.3	56.7	46.3	53.7	45.9	54.1	53.0	47.0	56.0	44.0	71.1	28.9	76.4	23.6	78.5	21.5
Social sciences, humanities, arts	86	59.3	40.7	60.5	39.5	60.5	39.5	53.5	46.5	74.4	25.6	79.1	20.9	77.9	22.1	79.1	20.9
Engineering, math, computer science, unknown	50	60.0	40.0	61.2	38.8	64.0	36.0	68.0	32.0	70.0	30.0	92.0	8.0	82.0	18.0	80.0	20.0
*Parental/guardian education*																	
Parents/guardians are not college educated	118	41.5	58.5	48.3	51.7	44.4	55.6	48.3	51.7	57.6	42.4	67.8	32.2	72.6	27.4	77.1	22.9
Parents/guardians are college educated	169	57.7	42.3	56.6	43.4	59.9	40.1	61.1	38.9	68.5	31.5	83.8	16.2	81.4	18.6	80.2	19.8
*Pell Grant eligible*																	
Not eligible for Pell Grant	160	50.9	49.1	56.7	43.3	56.1	43.9	58.9	41.1	67.3	32.7	83.5	16.5	81.1	18.9	80.4	19.6
Federal Pell Grant eligible	127	51.2	48.8	48.8	51.2	50.4	49.6	52.0	48.0	59.8	40.2	69.3	30.7	73.6	26.4	77.2	22.8
*First-generation college student*																	
Not a first-generation college student	193	54.7	45.3	55.8	44.2	57.1	42.9	59.7	40.3	66.7	33.3	84.3	15.7	81.2	18.8	79.1	20.9
First-generation college student	94	43.6	56.4	47.9	52.1	46.2	53.8	47.9	52.1	58.5	41.5	62.8	37.2	71.0	29.0	78.7	21.3

Binary logistic regressions were performed to provide more detailed multivariate analyses. A multiple regression analysis is conducted for each dependent variable. All the independent variables are placed in the model in a forward stepwise fashion; only the ones that had significant effects on the dependent variable are retained in the final model. These modeling techniques are similar to the ones used in Bunyan et al. (2016).

Tables 5 and 6 present the final models. For each explanatory variable shown in the tables, the value of the coefficient, standard error (S.E.), Wald chi-square (χ^2^) statistic, the significance level or *ρ*-value, and the odds ratio (Exp(β)) are included. The results of the Cox and Snell, as well as Nagelkerke pseudo-R-Square analysis, are presented; these are used to evaluate the goodness of fit of the models. The classification rate—which indicates the percentage of cases that the models predict correctly—is included for each model. The tables also list the excluded (or reference) category for each explanatory variable in the models.

**Table 5:** Logistic regression models 1–4 for respondents' thoughts about natural hazards, disconnection, predators, and getting lost

Variables	Model 1. Thought about natural hazards					Model 2. Thought about being disconnected				
	*β*	S.E.	Wald χ^2^	*p*-value	Exp(*β*)	*β*	S.E.	Wald χ^2^	*p*-value	Exp(*β*)
Age^a^	0.941	0.372	6.409	0.011	2.562					
Parent/guardian education^b^	−0.702	0.253	7.722	0.005	0.495					
Major^c^			8.744	0.013				7.479	0.024	
Social sciences, humanities, arts	−0.657	0.282	5.424	0.020	0.519	−0.671	0.286	5.498	0.019	0.511
Engineering, math, computer science, unknown	−0.849	0.348	5.940	0.015	0.428	−0.712	0.348	4.178	0.041	0.491
Gender^d^						−0.747	0.298	6.261	0.012	0.474
Race^e^								8.345	0.039	
Asian						0.743	0.339	4.800	0.028	2.102
Black or African American						0.702	0.342	4.222	0.040	2.017
LatinX/other						0.827	0.341	5.872	0.015	2.285
Constant	0.585	0.239	6.015	0.014	1.795	0.279	0.336	0.693	0.405	1.322
R^2^ Cox–Snell	0.077					0.074				
R^2^ Nagelkerke	0.103					0.098				
Classification (%)	60.8					66.5				
*N*	286					284				

Variables	Model 3. Thought about predators					Model 4. Thought about getting lost				
	*β*	S.E.	Wald χ^2^	*p*-value	Exp(*β*)	*β*	S.E.	Wald χ^2^	*p*-value	Exp(*β*)
Race^e^			16.844	0.001				14.786	0.002	
Asian	0.747	0.339	4.862	0.027	2.110	1.005	0.335	9.012	0.003	2.731
Black or African American	1.401	0.350	15.997	0.000	4.058	1.008	0.338	8.901	0.003	2.740
LatinX/other	0.808	0.343	5.563	0.018	2.243	1.043	0.340	9.430	0.002	2.837
Major^c^			9.053	0.011						
Social sciences, humanities, arts	−0.722	0.288	6.256	0.012	0.486					
Engineering, math, computer science, unknown	−0.825	0.350	5.552	0.018	0.438					
Constant	−0.424	0.234	3.300	0.069	0.654	−0.909	0.220	17.096	0.000	0.403
R^2^ Cox–Snell	0.085					0.053				
R^2^ Nagelkerke	0.114					0.071				
Classification (%)	63.7					59.3				
*N*	284					285				

Reference categories used in the models:

^a^16–24 years old

^b^Parents/guardians are not college educated

^c^Physical and natural sciences

^d^Male

^e^White or Caucasian (not LatinX)

**Table 6:** Logistic regression models 5–8 for respondents' thoughts about man-made hazards, loathesome places, fearfulness, and danger

Variables	Model 5. Thought about Human-made hazards					Model 6. Thought about loathsome, hateful places				
	*β*	S.E.	Wald χ^2^	*p-*value	Exp(*β*)	*β*	S.E.	Wald χ^2^	*p-*value	Exp(*β*)
Parent/guardian education^a^	−0.522	0.256	4.167	0.041	0.593					
Major^b^			9.483	0.009				11.163	0.004	
Social sciences, humanities, arts	−0.850	0.299	8.054	0.005	0.427	−0.603	0.350	2.976	0.085	0.547
Engineering, math, computer science, unknown	−0.676	0.354	3.633	0.057	0.509	−1.832	0.580	9.974	0.002	0.160
Gender^c^						−0.729	0.355	4.216	0.040	0.483
Race^d^								14.879	0.002	
Asian						1.249	0.496	6.350	0.012	3.488
Black or African American						1.547	0.485	10.156	0.001	4.698
LatinX/other						1.776	0.479	13.751	0.000	5.909
First-generation college student^e^						−0.938	0.319	8.632	0.003	0.391
Constant	0.076	0.226	0.112	0.738	1.079	−0.854	0.534	2.561	0.109	0.426
R^2^ Cox–Snell	0.045					0.160				
R^2^ Nagelkerke	0.062					0.243				
Classification (%)	64.7					76.1				
*N*	286					285				

Variables	Model 7. Thought about fear					Model 8. Thought about danger				
	*β*	S.E.	Wald χ^2^	*p-* value	Exp(*β*)	*β*	S.E.	Wald χ^2^	*p-* value	Exp(*β*)
Race^d^			8.083	0.044				10.820	0.013	
Asian	0.563	0.418	1.809	0.179	1.755	1.213	0.438	7.679	0.006	3.363
Black or African American	0.695	0.414	2.815	0.093	2.003	1.257	0.439	8.210	0.004	3.516
LatinX/other	1.122	0.398	7.967	0.005	3.071	1.197	0.443	7.298	0.007	3.309
Constant	−1.815	0.288	39.675	0.000	0.163	−2.208	0.333	43.937	0.000	0.110
R^2^ Cox–Snell	0.029					0.044				
R^2^ Nagelkerke	0.045					0.069				
Classification (%)	77.8					78.9				
*N*	284					285				

Reference categories used in the models:

^a^Parents/guardians are not college educated

^b^Physical and natural sciences

^c^Male

^d^White or Caucasian (not LatinX)

^e^Not a first-generation college student

### Limitations of the Study

The study has some limitations. The sample size is small. However, it is well within the range of samples used in this type of research. The study also focuses on a specific population rather than the general population. Because of time and funding constraints, it was not feasible for the researcher to obtain a national randomized sample.

The study uses a purposive sampling technique. Such a method is subjective because the researcher relies on his or her knowledge of the subject area, experience, and judgment in the sample-selection process (Guarte and Barrios 2006). Despite the limitations of the purposive sampling technique, Guarte and Barrios (2006) conclude that this approach can yield the reliable results. The limitations mentioned above can limit the replicability of the study and the generalizability of the results.

## Results

### Contemplating Nature

When respondents think about nature, they are most likely to think about the outdoors, trees and forests, as well as bodies of water. As Table 1 shows, more than 90% of the sample think of the outdoors, trees, forests, plants, green space, and animals frequently when they think of nature. While 81.1% think of remote and far away landscapes, and 66.2% think of rural landscapes. Only 29.1% think of urban landscapes frequently. Moreover, two-thirds or more of the students thought about the utility of nature frequently when contemplating nature. Study participants also thought about the sensations and feelings nature evokes as well as their experiences in nature. That is to say, more than 60% of the sample say they have these thoughts regularly.

Study participants were far more likely to think about the above themes rather than fear/danger/loathing. Overall, respondents gave the lowest scores to these ideas. All eight concepts had mean scores of less than 2.75. In other words, between 51 and 79% of the study participants said they think of these factors infrequently when they contemplate nature.

### Natural Hazards

Table 4 shows that between 47 and 52% of whites, Asians, blacks, and LatinX/others say they think about natural hazards frequently when they think of nature. Males were more likely than females to think about natural hazards frequently; 56.9% of males and 46.6% of females said they did. Older students were more likely to think of natural hazards frequently than those who were under 25 years of age. While 61.1% of graduate students thought about natural hazards frequently, only 46.1% of undergraduates did. Roughly, 57% of natural/physical science majors thought of natural hazards often; this far exceeds the 40–41% of other students who think of this factor frequently.

Respondents whose parents/guardians did not attend college were much more likely to think of natural hazards frequently than participants whose parents/guardians are college educated. Hence, 42.3% of those with college-educated parents/guardians and 58.5% of those whose parents/guardians did not attend college thought about natural hazards frequently. Regarding natural hazards, the difference between respondents who were eligible for Pell Grants and those who were ineligible, was inconsequential. However, students who were first-generation college students were more likely to report that they thought about natural hazards frequently than those who were not first-generation college students.

Three of the explanatory variables were significant in the multivariate model (see Table 5, Model 1). The *R*^*2*^ values are between 0.077 and 0.103, and the case classification rate is 60.8%. The regression shows that age is significant (χ^2^ = 6.409, *ρ* = 0.011), and is the first variable retained in the model. The odds ratio indicates that respondents who are 25 years or older are 2.562 times more likely to think about natural hazards frequently than younger participants. Parental/guardian education is another independent variable that is significant (χ^2^ = 7.722, *ρ* = 0.005). Students with college-educated parents/guardians are 0.495 times less likely to think of natural hazards frequently than those without college-educated parents/guardians.

The other independent variable that had a significant effect on the final model was academic major (χ^2^ = 8.744, *ρ* = 0.013). The model shows that students majoring in social science/humanities/arts were 0.519 times less likely, and those majoring in engineering/math/computer science/unknown were 0.428 times less likely to think about natural hazards frequently than natural/physical science majors.

### Disconnected

White students are less likely than ethnic/racial minority students to say they think about being disconnected from nature frequently (Table 4). While 35.6% of white respondents thought about being disconnected frequently, 51.6–55% of the remaining students did likewise. Males were significantly more likely than females to think about disconnection from nature. Hence, 60.1% of males and 42.7% of females frequently thought about being disconnected from nature. Older respondents are slightly more likely than younger participants to report thinking about disconnectedness from nature often.

Graduate students have a greater tendency to think of disconnection from nature than undergraduates. That is, 57.4% of graduate students and 44.3% of undergraduates often think of being disconnected from nature. While 53.7% of the natural/physical science majors think about disconnection from nature frequently, roughly 38% of other students do the same. Respondents whose parents did not attend college, those who are Pell Grant eligible, as well as first-generation college students are more likely to think of being disconnected from nature frequently than other students.

Three independent variables—gender, age, and academic major—were significant in the regression model (see Table 5, Model 2). The *R*^*2*^ values are 0.074 and 0.098; the case classification rate is 66.5%. Gender had a significance level of χ^2^ = 6.261 and *ρ* = 0.012. The Exp(β) indicates that females were 0.474 times less likely than males to think about disconnection frequently when they cogitate nature. Asians, blacks, and LatinX/others were all more than twice as likely as whites to think regularly about disconnection from nature. Social science, humanities, and arts majors were 0.511 times less likely than natural/physical science students to think about disconnection from nature regularly. In comparison, engineering/math/computer science/unknown majors were 0.491 times less likely than natural/physical science majors to think about disconnectedness frequently.

### Predators

Blacks stand out as having a much higher propensity to think about predators when reflecting on nature than other respondents (Table 4). Consequently, 63.9% of blacks regularly thought about predators when contemplating nature, while 32.7% of whites did similarly. In comparison, 47.6% of Asians and 50.8% of LatinX/other respondents considered predators frequently when they thought of nature.

There is a relationship between academic interests and the probabilities of thinking about predators when considering nature. To wit, natural/physical science students are much more apt to think of predators than other students. Hence, 54.1% of natural/physical science respondents often think of predators when cogitating nature. In contrast, 39.5% of social science/humanities/arts majors and 36% of engineering/math/computer science/unknown majors focus on predators often while contemplating nature.

Males and females think about predators similarly. Older respondents and graduate students are more liable to think of predators than those who are under 25 years of age or who are undergraduates. As was the case with disconnectedness, respondents whose parents/guardians did not attend college, those who qualify for Pell Grants, and first-generation college students are more inclined to ponder predators often when considering nature than students with college-educated parents/guardians, who are not eligible for Pell Grants, or who are not first-generation college attendees.

Only two independent variables—race and academic major—are significant in the multivariate regression (Table 5, Model 3). The *R*^*2*^ values are 0.085 and 0.114, while the case classification rate for the model is 63.7%. Race is very significant and contributes the most to the final model (χ^2^ = 16.844, *ρ* = 0.001). The odds ratio signifies that while Asians and LatinX/others are just over two times more likely than whites to think about predators frequently when imagining nature, blacks are 4.058 times more inclined to think about predators in this context than whites.

The significance level for academic major is χ^2^ = 9.053 and *ρ* = 0.011. The odds ratio indicates that social science/humanities/arts majors were 0.486 times less likely and engineering/math/computer science/unknown majors 0.438 times less prone to think about predators frequently than natural/physical science students when they reflect on nature.

### Getting Lost

A much lower percentage of white students reported that they regularly think about getting lost when they reflect on nature than racial/ethnic minority students. While 28.7% of white respondents often think about getting lost, more than half of the other study participants considered this factor regularly.

Males were only slightly more inclined to think of getting lost than females. Similarly, when respondents who were 25 years and older reflected on nature, they were only slightly more disposed to think of getting lost frequently than younger respondents. More than half of the undergraduates (57.6%) think of getting lost often. Conversely, 48.1% of graduate students do likewise. Just over half of the natural/physical science majors and social science/humanities/arts majors think about getting lost frequently, but 68% of engineering/math/computer science/unknown majors often think about getting lost when they reflect on nature.

Race was the only explanatory variable that had a significant effect on the multiple regression model (Table 5, Model 4). The *R*^*2*^ values were 0.053 and 0.071; the case classification rate for the model was 59.3%. The model had a χ^2^ of 8.744 and a *ρ*-value of 0.013. The odds ratio signifies that Asians were 2.731 times, blacks 2.740 times, and LatinX/others 2.837 times more inclined to think about getting lost on a regular basis when they reflect on nature than whites were.

### Human-made Hazards

Asians (27%) were the least likely and LatinX/others (48.3%) the most prone to think about human-made hazards frequently when they consider nature (Table 4). On the other hand, similar percentages of blacks (37.1%) and whites (33.7%) contemplated human-made hazards regularly. A higher percentage of males thought about human-made hazards often than females. Older respondents also had a higher propensity to reflect on human-made hazards regularly than participants who were under the age of 25 years of age.

While 46.3% of graduate students said they reflected on human-made hazards often, a third of the undergraduates thought about this factor regularly. A higher percentage of students who were natural/physical science majors tended to think about human-made hazards regularly than their peers. The pattern observed earlier with other factors held for this dependent variable too. That is, respondents whose parents are not college educated, those who qualify for Pell Grants, and first-generation college students are more apt to think about human-made hazards frequently than other students. However, in this instance, parental/guardian education is significant. While 42.4% of the students whose parents/guardians did not attend college thought about human-made hazards frequently, 31.5% of the respondents with college-educated parents thought about this factor frequently when they thought of nature.

The logistic regression for this factor appears in Table 6, Model 5. Parental/guardian education and academic major make significant contributions to the final model. The case classification rate is 64.7%, and the *R*^*2*^ values are 0.045 and 0.062. Parental/guardian education has a χ^2^ of 4.167 and *ρ-*value of 0.041. According to the odds ratio, respondents with college-educated parents/guardians were 0.593 times less likely than those with parents/guardians who did not go to college to think frequently about human-made hazards. The χ^2^ for student's academic major was 9.483, and the *ρ*-value was 0.009. The Exp(β) shows that social science/humanities/arts majors were 0.427 times and engineering/math/computer science/unknown majors were 0.509 times less probable than natural/physical science majors to think of human-made hazards frequently when reflecting on nature.

### Loathsome, Hateful Places

Only 7.9% of white students think of loathsome, hateful places frequently when they consider nature. However, significantly higher percentages of ethnic/racial minority students make this association with nature. Hence, 23.4% of Asians, about a third of blacks, and 36.7% of LatinX/others frequently think of hateful or loathsome places when they cogitate nature.

Almost 31% of males think of loathsome, hateful places regularly when they contemplate nature. In contrast, only 20.5% of females do likewise. Whereas 31.7% of respondents who were 25 years or older envisioned loathsome, hateful places often when thinking of nature, 21.3% of younger study participants think similarly.

Graduate students were more prone to conjure up images of loathsome, hateful places often when thinking of nature than undergraduates. Only 8% of engineering/math/computer science/unknown majors often thought about loathsome, hateful places when reflecting on nature. In comparison, 20.9% of the social science/humanities/arts majors and 28.9% of the natural/physical science majors had similar thoughts. First-generation college students had a significantly higher tendency to think about loathsome, hateful places frequently when considering nature than their peers who were not the first generation in their families to attend college. Hence, 37.2% of first-generation and 15.7% of non-first-generation college students thought about this factor frequently.

Academic major, race, gender, and first-generation college students were the explanatory variables that had significant effects on the final regression model (Table 6, Model 6). The model had a case classification rate of 76.1% and *R*^*2*^ of 0.160 and 0.243. Regarding academic major, the χ^2^ was 11.163, and the *ρ-*value was 0.004. Social science/humanities/arts majors were 0.547 times less disposed than natural/physical science majors to think of loathsome, hateful places frequently when thinking of nature. However, engineering/math/computer science/unknown majors were only 0.160 times less likely than natural/physical science majors to do likewise.

The odds ratio indicate that females were 0.483 times less disposed than males to invoke images of loathsome, hateful places regularly when contemplating nature. This variable has the following significance leve: χ^2^ = 4.216 and *ρ* = 0.040. Race is very significant in the final model (χ^2^ = 14.879, *ρ* = 0.002). The Exp(β) signifies that Asians were 3.488 times, blacks 4.698 times, and LatinX/others 5.909 times more likely than white respondents to think of loathsome, hateful places frequently when they think of nature.

The intergenerational impact of college attendance is also significant in this model. That is, non-first-generation college students are 0.391 times less likely than first-generation college respondents to envision loathsome, hateful places frequently when thinking about nature. The significance level for this variable is χ^2^ = 8.632 and *ρ* = 0.003.

### Fearful

LatinX/other respondents were the most and white students the least apt to say that they thought about fear frequently when they contemplated nature (Table 4). While a third of LatinX/other students envisioned fear often when they thought about nature, only 14% of white participants did. However, Asians and blacks thought somewhat similarly about fearfulness and nature. That is, 22.2% of Asians and 26.4% of blacks thought about fear regularly when reflecting on nature.

There is a greater tendency for males to think about fearfulness frequently when nature comes to mind than females. As was the case with other factors examined above, graduate students (25.9%) were slightly more prone to conjure up fear often when thinking of nature than undergraduates (21.3%).

Eighteen percent of engineering/math/computer science/unknown majors think about fear in connection to nature regularly; only a slightly higher percentage of social science/humanities/arts majors (22.1%) and natural/physical science majors (23.6%) responded similarly. The differences in categorical responses for the variables parental/guardian education, Pell Grant eligibility, and first-generation college adhered to the pattern identified earlier.

Race was the only explanatory variable that was significant in the multivariate model (Table 6, Model 7). The *R*^*2*^ values were 0.029 and 0.045. The case classification rate of the model was 77.8%. It had a χ^2^ of 8.083 and a *ρ-*value of 0.044. The odds ratio indicates that Asians were 1.755 times, blacks 2.003 times, and LatinX/others 3.071 times more likely than whites to think of fear frequently when reflecting on nature.

### Dangerous

Respondents tended to think similarly about danger when they think of nature. Race was the only independent variable that manifested significant variations in the categories of danger analyzed. A low percentage of white students said that they thought about danger frequently when they think of nature; 9.9% of them did this. Ethnic/racial minorities were very consistent in their responses—roughly 27% of them envisaged danger often when they thought about nature.

While 27.7% of males imagined danger regularly when they considered nature, only 19.1% of females did likewise. Younger respondents were more inclined to think of danger frequently when they cogitated nature than those 25 years and older. Similarly, undergraduates were more apt to envision danger often when they contemplate nature than graduate students.

Students in different academic majors were virtually identical in the way they thought about danger and nature. Respondents whose parents/guardians did not attend college, those who qualify for Pell Grants, and first-generation college students were slightly more likely than their counterparts to think about danger frequently in association with nature.

Only one variable—race—had significance in the regression model (Table 6, Model 8). The case classification rate was 78.9%, and the *R*^*2*^ values were 0.044 and 0.069. The χ^2^ was 10.820, and the *ρ*-value was 0.013. The odds ratio shows that Asian, black, and LatinX/other respondents were 3–3.5 times more likely than white students to envision danger frequently when they think about nature.

## Recap, Discussion, and Conclusions

The findings of this research demonstrate the need for scholars who study connections to and disconnections from nature to expand the repertoire of explanatory variables used to examine these phenomena. It is clear from this study that educational, generational, age, and class variables influence thoughts about nature. Culture, rurality, urbanization, region, and nationality are additional variables that could influence such thoughts. Although scholars have dedicated much effort to studying the relationship between race and nature, this paper suggests that the intense focus on blacks and whites misses essential nuances that can help us understand people’s perceptions of and relationship to nature more fully.

So, what matters in the way people think about nature? Six of the independent variables studied had significant impacts on the dependent variables in the multivariate models. Box 4 summarizes these impacts. The study found that race had significant effects on thinking about disconnectedness, predators, getting lost, loathsome and hateful places, fearfulness, and danger in the context of reflecting on nature. However, researchers should not stop there. Scholars should not think of racial groups as monolithic entities or think that race is the be all and end all to understanding human perceptions of nature. This study indicates that not all members of a particular racial or ethnic group think about nature in the same manner. Consequently, managers should refrain from making too many generalizations about any given racial group when it comes to their thoughts about, understanding of, and interactions with nature.

### Box 4. Summary of significant effects in the multivariate models

1. Race
  - Disconnectedness
  - Predators
  - Getting lost
  - Loathsome and hateful places
  - Fearfulness,
  - Danger
2. Academic Major
  - Natural hazards
  - Disconnectedness
  - Predators
  - Human-made hazards
  - Loathsome and hateful places
3. Gender
  - Disconnectedness
  - Loathsome and hateful places
4. Parent/Guardian Education
  - Natural hazards
  - Human-made hazards
5. Age
  - Natural hazards
6. First-Generation College
  - Loathsome and hateful places

As the results of this study have shown, a variable—students’ academic interests—often overlooked in this type of research, also had significant influences on five dependent variables. Academic majors had significant impacts on thinking about natural hazards, disconnectedness, predators, human-made hazards, and loathsome or hateful places. Gender was important in thinking about disconnectedness as well as loathsome/hateful places, whereas parent/guardian’s educational attainment had significant influences on thinking about both natural and human-made hazards. Age was influential in thinking about natural hazards while being a first-generation college student had significant effects on thinking about loathsome and hateful places.

Though parental/guardian educational attainment is significant in two multivariate models, the student’s educational attainment was not significant in any of the models. Another explanatory variable—Pell Grant eligibility—was not retained in any of the multiple regression models either. Though these two independent variables were not influential in this study, they might be of significance when researchers explore other dimensions of thinking about nature.

This study suggests some important things about social class that is not described in earlier studies in this genre. That is, being low income (as measured by Pell Grant eligibility) did not have significant effects on thoughts about nature. However, the educational attainment of one’s parents/guardians did. Hence, having parents/guardians who were not college educated was associated with more frequent thoughts about natural hazards and human-made hazards. In addition, another income indicator, first generation in college, was significant in respondents regularly thinking of nature as loathsome and hateful places. These findings lend credence to the intergenerational transmission thesis posited by Hyun (2005) and Gotch and Hall (2004).

Although race is salient in understanding people’s thoughts about nature, the effects do not necessarily manifest themselves in the way previous scholarship has suggested. Researchers have reported that blacks exhibit an aversion to the wilds, are fearful of natural settings, and are disconnected from nature (Kellert 1984; Schroeder 1989; Johnson 1998; Lewis and Hendricks 2006; Talbot and Kaplan 1984; Brownlow 2006; Floyd et al. 1995).

The findings of this study show that thoughts about fearing nature, danger in nature, and disconnection from nature are not exclusive or unique to blacks—all racial groups expressed these sentiments. Besides, 47.5% of blacks think of being disconnected from nature infrequently. Furthermore, most blacks do not think of nature as loathsome. That is, two-thirds of blacks associate nature with hateful or loathsome places occasionally, and 72.1% think of danger infrequently when they reflect on nature. Similarly, high percentages of Asians and LatinX/other respondents think of danger and hateful or loathsome places infrequently when they think of nature. It is true that there are significant differences in the way whites think about these aspects of nature and the way racial/ethnic minorities do, but we should not lose sight of the fact most minority college students are not always fearful of or disconnected from nature when they think of it.

Unlike earlier studies that find that females consider the environment to be riskier than males (Kellstedt et al. 2008; van der Linden 2015; Bunyan et al. 2016), this study does not fully support those findings. Findings from this study show that male students had a higher propensity than females to think of natural and human-made hazards, getting lost, fear, and danger regularly when they reflected on nature. Though Manning (2012) argues that males are more connected to nature than females, the findings of this study would not necessarily lead one to reach this conclusion.

This study suggests that environmental managers should think harder about the following question: What is the relationship between what people think about nature and the way they act in concert with nature? In other words, what is the relationship between thoughts, perceptions, and action? This study implies that managers should connect these factors more deliberately in their planning and programming.

This study reveals significant findings related to a specific segment of the adult population— college students. College students are highly educated and tend to be efficacious and action oriented. The data presented above show that, regardless of race, college students generally think about the favorable aspects of nature frequently. College students' generally positive view of nature is a useful insight for managers to consider; as such, students are influencers who can act as supporters of nature and as liaisons between managers and the general public. College students can help to activate and spread support for nature among the general public. They can also be a bridge to reaching underserved populations. Buijs and Elands (2013) believe that understanding the way people think about nature “may improve communication and collaboration between professionals and stakeholders.” Working with influencers who can communicate on multi-media platforms could be quite helpful to environmental managers as there is an urgent need to reach broader audiences and incorporate more historically underrepresented people in the planning for and management of natural areas. Managers could benefit from the insights that people who are not typically involved in environmental management might have.

Environmental managers should also embrace the flowering of people-of-color-led nature-focused groups that have materialized over the last decade. These groups, organized and operated mostly by college-educated millennials, include organizations, such as Latino Outdoors; Outdoor Afro; Asian Outdoors; Natives Outdoors; People of the Global Majority in the Outdoors, Nature, and Environment; Trail Posse; and Brown Girls Climb. Environmental managers should collaborate with groups like these to develop culturally sensitive curricular materials and outdoor education activities. Environmental managers should also include leaders of these organizations in policy and planning, employ members of these organizations, and fund opportunities that increase access to natural areas to program participants.

Though the study provides critical new insights into college students’ thoughts about nature, there are limits to the extent to which one can generalize from the results. Not only does this study focus only on a specific subset of the young adult population but students themselves are also very diverse, and this study does not capture the full range of that diversity.

There is a need for more research on this topic. This study suggests that we should try to find out how people think about different types of nature. That is, how do they think about woodlands, forests, savannahs, alpine meadows, swamps, and pastoral landscapes, to name a few. We need to examine concepts such as object-specific and situational fear more thoroughly in future research. It would help environmental managers if they knew whether fear of a particular object or situation that arises in nature grows into generalized anxiety that compromises a person’s perception of and feelings towards nature.
